# Political commitment and implementation: the health system response to violence against women in Mexico

**DOI:** 10.1093/heapol/czaf012

**Published:** 2025-03-08

**Authors:** Sophie M Morse, Manuela Colombini, Erin C Lentz, Carolina Dotoreo Soriano, Ana Jocelyn Mejía Morales, Claudia Diaz Olavarrieta, Daniela C Rodríguez

**Affiliations:** Philip R. Lee Institute for Health Policy Studies, University of California, 490 Illinois Street, San Francisco, CA 94158, United States; Department of Global Health and Development, London School of Hygiene & Tropical Medicine, Keppel Street, London WC1E 7HT, United Kingdom; The Lyndon B. Johnson School of Public Affairs, The University of Texas at Austin, 2315 Red River Street, Austin, TX 78712, United States; Facultad de Medicina, Universidad Nacional Autónoma de México, Escolar 411A, Copilco Universidad, Mexico City 04360, Mexico; Facultad de Medicina, Universidad Nacional Autónoma de México, Escolar 411A, Copilco Universidad, Mexico City 04360, Mexico; Facultad de Medicina, Universidad Nacional Autónoma de México, Escolar 411A, Copilco Universidad, Mexico City 04360, Mexico; Department of International Health, Johns Hopkins Bloomberg School of Public Health, 615 N Wolfe Street, Baltimore, MD 21205, United States

**Keywords:** violence against women, policy implementation, Mexico, health policy

## Abstract

Violence against women (VAW) is a widespread concern globally and in Mexico, where in 2021, 44% of women aged ≥15 years have experienced some form of intimate partner violence during their relationship and a quarter have experienced sexual violence in the past 12 months. To respond, Mexico passed comprehensive legislation addressing VAW, which outlines the role of the health system in identifying, treating, and referring women experiencing violence; however, implementation of such regulations has been slow and far from successful. Using a conceptual framework of political commitment, we conducted a health policy analysis to evaluate how health policies addressing VAW in Mexico have been implemented. The political commitment framework includes the dimensions of expressed, institutional, and budgetary commitment. We adopted a multi-methods qualitative case study approach combining document analysis with 25 stakeholder interviews with policymakers and health facility directors in Mexico City. The results show that Mexico exhibits limited expressed political commitment from the president, but some commitment exists among Ministry of Health officials. We document that the mixed findings on expressed commitment are mediated, in part, by internalized social and gender norms that normalize and tolerate violence, which are present in society writ large. We find that institutional commitment exists through policies and institutions. However, monitoring systems for policy implementation were not working as designed, and there was limited effort and capacity to implement these policies, reflecting structural barriers and norms within the health system that shape the treatment of violence survivors. Finally, we found a budgetary allocation for VAW; however, it was unclear if the budget was utilized correctly. While progress has been made in addressing VAW in the health system in Mexico, implementation is lagging due to a lack of sustained political commitment, and thus, policies are likely not reaching their intended beneficiaries: survivors of violence.

Key messages

- Violence against women (VAW), rooted in gender inequality, remains a pervasive public health problem globally and in Mexico, with health and social consequences for those who experience it, their children, families, and communities.
- Mexico has a comprehensive policy and legal framework to address VAW, which outlines the role of the health system, but successful implementation requires strategies that go beyond legislation.
- Political commitment is a useful framework to identify macro-level factors influencing the implementation of policies addressing VAW, shedding light on how political commitment facilitates or inhibits implementation in the dimensions of expressed, institutional, and budgetary commitment.
- In Mexico, there are mixed levels of political commitment to address VAW due to insufficient expressed commitment, institutional limitations, budgetary intricacies, and deeply rooted societal norms on *machismo*, which perpetuate tolerance toward violence and mediate the relationship between the dimensions of political commitment and implementation.

## Introduction

Violence against women (VAW) is a human rights and global public health problem leading to numerous short- and long-term health, economic, and social consequences for the people who experience it, their children, and their communities (Krug et al. 2002, García-Moreno et al. 2015a, World Health Organization 2021a). One in three women globally has experienced some form of VAW, with 26% of women aged ≥15 years having experienced physical and/or sexual violence from a current/former husband or a male intimate partner at least once in their lifetime (World Health Organization 2021a). In post-colonial patriarchal contexts, there is a heightened risk for intimate partner violence (IPV), highlighting how gender inequality and colonial history drive high rates of IPV globally (Brown et al. 2023). IPV is a complex social problem rooted in unequal power relations between men and women, requiring a multi-sectoral response. Due to its myriad consequences, there is global recognition of the critical role of the health system in this response (World Health Organization 2013, 2016, World Health Organization 2014).

Global experts contend that it is possible to eliminate VAW and violence against girls. For this to occur, political commitments must be translated into meaningful change and actions by governments, communities, and civil society partners (García-Moreno et al. 2015a). A recent Pan American Health Organization report shows that in the Americas—the focus of this study—80% of countries have a multi-sectoral VAW plan, while 83% have a health policy that mentions VAW (Pan American Health Organization 2022). Despite critical progress, policies and plans must be translated into supportive health services, and the implementation status is less well known (World Health Organization 2021b, Pan American Health Organization 2022).

In Latin America, interpersonal violence is often gendered, reinforcing and/or taking advantage of a gendered *status quo* (Weitzman et al. 2024). Following Weitzman et al.’s criteria, interpersonal violence refers to violence where a perpetrator is motivated by the prospect of their own gains, the involved parties are socially connected to one another and/or the perpetrator uses their discretion about when and how to behave violently (sometimes going against a group or organizational directive) (Weitzman et al. 2024). In Mexico, there are high levels of VAW, one form of interpersonal violence, with 44% of women experiencing IPV in their lifetime and nearly 20% experiencing sexual violence in the past 12 months (INEGI 2022). There are 10 recorded femicides a day, but the actual numbers are likely much higher due to issues with how these deaths are classified from state to state (Sandin 2020, Data Cívica, Intersecta 2021).

Mexico also has some of the highest rates of intentional homicide in Latin America (28.2/100 000) (Flores Martínez and Phillips 2022, United Nations Office on Drugs and Crime 2023). Widespread violence, largely attributable to drug trafficking and organized crime, disproportionately affects men (Heinle et al. 2017) and can also become interpersonal when it goes beyond violence exerted as directives from criminal organizations or drug traffickers and is not intended to further the aims of any formally defined group or cause (Weitzman et al. 2024). In some parts of Latin America, male-to-male violence has been shown to increase the likelihood of IPV (Kiss et al. 2015), highlighting how multiple forms of interpersonal violence reinforce each other (Weitzman et al. 2024).

Mexican gender relations have also been profoundly shaped by the Catholic church’s effort to maintain its power over conservative cultural discourse and moral politics, fomenting religious conceptions of women’s identities that lead to discrimination and reinforcement of a culture of *machismo* that defines rigid gender roles by upholding men’s patriarchal privileges and women’s subjugation (Blofield 2013, Heep 2014). There is a “cultural tolerance” of male social dominance and patriarchy as they are accepted as part of Mexican culture, with the associated values of dominance, control, and protectiveness (Nieto 2020).

Nevertheless, over the past 30 years, Mexico has made significant progress in addressing VAW. The formulation of policies is a socio-political process (Koduah et al. 2016, Chipendo et al. 2021), and when governments or institutions develop policies, they endorse specific values, prioritizing them over others in health system decisions (Kenny and Giacomini 2005, Vélez et al. 2020). In Mexico, VAW was first recognized publicly as a social problem in the 1970s by feminist activists and legislators, which paved the way for eventual legislation on this issue (Schneider and Ingram 1993, Koon et al. 2016, Beer 2017, Lamas et al. 2018).

Mexico ratified the Convention on the Elimination of All Forms of Discrimination Against Women (CEDAW) in 1981 and the Inter-American Convention on the Prevention, Punishment, and Eradication of Violence Against Women (“*Belém do Pará*”) in 1998 (OHCHR *n.d*.; Organization of American States 1994). In 2007, landmark legislation was passed: the General Law for Women’s Access to a Life Free from Violence (hereby the “General Law”). It established a legal framework articulating women’s rights to live free of violence and discrimination and to have free health-care and other services (Cámara de Diputados 2007).

The Mexican federal Ministry of Health (MoH) has also adopted a VAW policy framework to guide the health system response. In 2005, it published the Official Mexican Norm titled Family, Sexual Violence and Violence Against Women Guidelines for Prevention and Services (a norm in Mexico is an official, compulsory regulation) (Estados Unidos Mexicanos-Secretaría de Salud 2005). Among other supportive services, the MoH established a Specialized Services program to offer care and psychosocial support to VAW survivors in health facilities (Centro Nacional de Equidad de Género y Salud Reproductiva 2022a).

Despite the VAW policy and legal framework, the implementation of policies and laws has been slow. A study by Casique Rodríguez (2015) using nationally representative data found that fewer women who had experienced violence sought institutional help in 2011 than in 2006 before the General Law was passed (22% in 2006 versus 10% in 2011). Similarly, the percentage of women considering separation—an indication that someone may be leaving an abusive partner—decreased (32% in 2006 versus 25% in 2011). These results suggest limited implementation of the law.

Regarding health service availability, only ∼16% of public health facilities in Mexico provide health services to women experiencing violence (Buenrostro et al. 2017). The Specialized Service program has not been appropriately monitored or evaluated due to a lack of indicators to measure its long-term success and inconsistencies in reporting from the program and health facilities regarding the number of patients served (Escalante Ferrer 2019). Moreover, public health institutions do not always detect and address violence cases, with evidence showing that women at domestic violence shelters have not been screened (Austrian Centre for Country of Origin and Asylum Research and Documentation (ACCORD) 2024; Lachenal et al. 2016). Research with health-care workers (HCWs) has also shown that sometimes survivors of IPV are blamed for the violence, face misogynistic comments, and have their credibility questioned (Herrera 2010). Women’s utilization of health services for VAW is very low, often due to shame, stigma, or lack of trust in health providers, and the percentage of women seeking health care has decreased over time (Frías 2013, Frías and Rios-Cázares 2017, INEGI 2022). Together, these findings indicate several barriers to implementing quality VAW health care (Buenrostro et al. 2017, Frías and Rios-Cázares 2017, Escalante Ferrer 2019).

While various studies have examined how laws on VAW in Mexico were passed, few have explored the implementation of policies addressing VAW in healthcare settings, and none with a focus on political commitment (Herrera 2010, Beer 2017, Buenrostro et al. 2017). Using Mexico as a case study, we use Fox et al.’s (2011) political commitment framework, which includes the dimensions of expressed, institutional, and budgetary commitments, to evaluate how policies to address VAW have been implemented and where shortfalls exist. In so doing, we endeavor to show how systematic analyses of policy implementation can aid in identifying ways to better support VAW survivors.

Political commitment has been recognized across health topics as necessary to draw attention to and, eventually, contribute to the development of health policies (Baker et al. 2018). While lots of theories and research have been published about why implementation is weak or fails, very few studies have looked at the role of continued political commitment, despite references to policy implementation failures citing the lack of political commitment as a rhetorical critique (De Leon 2021). Here, we leverage Fox et al.’s (2011) conceptual framework on political commitment to study implementation itself to unpack the extent to which the dimensions of expressed, institutional, and budgetary commitments play a role in the implementation process. We chose this framework to identify macro-level factors influencing implementation because in Mexico, a federalist state, if there is no sustained political commitment, then the policies, programs, and resources needed to respond to VAW at the health service/facility level are unlikely to be effectively implemented or sustained (Baker et al. 2019).

## Materials and methods

### Study context

Mexico was selected as the case for this study because it has many conditions for success and promising policies and programs, which enabled best practices to be studied (Yin 2009). Moreover, Mexico, like many other middle-income countries in Latin America and elsewhere, has a policy framework for addressing VAW on paper but is not fully implementing it (World Health Organization 2021b, Pan American Health Organization 2022). Given that the legal and policy framework to address VAW in Mexico was put in place almost two decades ago, the selection of this case was also based on the assumption that policy implementation should be well underway. In Mexico, VAW has typically been studied from a legal perspective, addressing delinquent individual behaviors that require legal action (Herrera 2010). We contribute to a growing body of literature on the health system’s response to this issue (Colombini et al. 2020, 2024, d’Oliveira et al. 2020, Sikder et al. 2021).

Interviews with key informants took place in Mexico City because the participants worked or had worked at central federal agencies located there. The capital city is considered one of Mexico’s most “advanced” states regarding gender legislation, including women’s health. Hence, besides allowing for access to federal policymakers, it was an appropriate place to identify some factors that facilitate and impede policy implementation at the state level.

### Study design

For this study, we drew on the field of health policy analysis, which embraces multiple methodologies and provides flexibility in the choice of framework (Walt et al. 2008). Accordingly, we adopted a multi-methods qualitative case study approach using qualitative interviews and document analysis. The document analysis was conducted before the interviews to provide context and inform the development of the topic guides. After reviewing various health policy analysis frameworks, we selected Fox et al.’s (2011) political commitment framework, described later, to frame the analysis and findings because political commitment is an essential driver of sustained action and implementation of policies and programs (Parkhurst and Lush 2004, Rodríguez et al. 2017).

### Conceptual framework of political commitment

We applied Fox et al.’s (2011) political commitment framework to study how policies on VAW were implemented in Mexico. This framework includes three key dimensions of political commitment: expressed commitment, institutional commitment, and budgetary commitment (outlined in Table 1) (Fox et al. 2011). The three dimensions of political commitment are shaped by determinants, effect modifiers, and mediators (Fox et al. 2011). Thus, political commitment is about the government’s intent and sustained actions to address an issue (Baker et al. 2018) and the factors influencing these dimensions. Political commitment has been used to analyze other public health issues (i.e. human immunodeficiency virus (HIV)/acquired immune deficiency syndrome, see Fox et al. 2011, Goldberg et al. 2012, and nutrition, see Baker et al. 2018), but it has yet to be applied to VAW. Furthermore, the political commitment framework can usefully unpack the gap between an issue getting on a policy agenda and its sustained implementation.

**Table 1.** Fox et al.’s (2011) political commitment framework

Dimension	Definition	Aspects of the dimension
Expressed commitment	Political support and perceived expressed commitment among policymakers	Policymakers’ express commitment to address VAW
Institutional commitment	Policy formulation, organizational structure, capacity of institutions, monitoring, and evaluation, as well as the legal and regulatory environment	Existence of policies addressing VAW
		When institutional structures (e.g. VAW commissions and centers) were established
		Presence of institutional structures/mechanisms that enable a response to VAW
Budgetary commitment	Allocation of resources	Resource allocation (i.e. budget line) for VAW

### Study methods

#### Document review

We used document analysis to systematically review policy documents, extracting data on some policies and budgets in place and providing essential context and historical insight into how VAW laws and policies were passed nationally (Creswell 2007, Bowen 2009). Fourteen policy documents were analyzed, including national policies, plans and frameworks, and budget documents (see Table 5 in the Supplementary material). A codebook based on the framework and study aim was used to conduct a thematic analysis of the content of policies (Mackieson et al. 2019).

#### Qualitative interviews

We conducted 25 key informant interviews with current and former national policymakers working on VAW, nongovernmental actors, and facility directors (of public hospitals and health centers in Mexico City). Interviewing policymakers is often called “elite interviewing” and differs from other interviews because of the power, authority, or status that people have (Van Audenhove and Donders 2019). We chose a mix of current and former policymakers because former policymakers can provide historical insight into implementation challenges, while current policymakers are often more reluctant to share criticisms of the government they work for due to their status and position. Facility director interviews were conducted at five health facilities in partnership with the Mexico City MoH. Participants were recruited purposively and through snowballing, as each was asked to recommend potential informants at the end of their interview. Potential key informants received information about the study and were followed up via email and WhatsApp.

Two interview guides were developed by respondent type. Policymakers were asked about policy implementation status, the institutions involved in implementation, aspects of political commitment, and the health sector’s role in responding to VAW. Facility directors were asked about priorities and norms within facilities as related to health services for survivors of violence. Interviews were conducted on Zoom (for COVID-19 safety reasons) or in person in Mexico City between September 2021 and June 2022. Interviews lasted 40–120 min. All interviews were conducted by the first author in Spanish and were recorded with consent. Recordings were transcribed and checked by Mexican research assistants for accuracy.

#### Data analysis

Transcripts were coded using thematic analysis (Guest et al. 2012). A deductive approach was used based on the political commitment framework. The sources of data for each dimension of the political commitment framework are outlined in Table 2. A codebook was developed based on the framework and was applied to all interviews. Documents and interviews were coded with Atlas.ti.

**Table 2.** Sources of information by dimension of political commitment

Dimension	Sources of information
Expressed commitment	Key informant interviews with current and former policymakers
Institutional commitment	Key informant interviews with current and former policymakers
	Document analysis of policies and plans
Budgetary commitment	Key informant interviews with current and former policymakers
	Document analysis of budget documents and reports

## Results

The results are presented around the three dimensions of the study’s conceptual framework. Participant characteristics are shown in Table 3.

**Table 3.** Participant characteristics

Key informants (*n* = 25)	
Gender	% (number)
Women	76% (19)
Men	24% (6)
Age	Mean: 44 years
Profession	
Lawyer	20% (5)
Psychologist	16% (4)
Doctor	28% (7)
Social scientist	16% (4)
Other	20% (5)
Relevant job/position	
Current policymaker	44% (11)
Former policymaker	28% (7)
Civil society	8% (2)
Facility director	20% (5)

### Expressed commitment

Our findings on expressed political commitment to address\VAW were mixed. Narratives from half of the policymakers showed that expressed commitment was lacking at the highest level: the presidency. These participants argued that the current president of Mexico (2018–24)—Andrés Manuel Lopez Obrador (hereafter AMLO)—has never centered women as a prominent part of his platform nor voiced a commitment to addressing VAW, explaining that throughout his long political career, women’s issues have never been a political priority. One participant shared, “Andrés Manuel does not adopt a gender perspective. He reproduces gender stereotypes implicit in society in his speeches” (Participant 6, civil society actor). Furthermore, one participant explained that it would be risky for AMLO to support sexual and reproductive health (SRH) issues, including VAW,

Beyond the opposition based on his own personal position, I believe that he sees sexual and reproductive health as a very risky issue in terms of political capital. There is violence, there is abortion, there is the issue of maternal death, right? That could be quite controversial in terms of the enforceability of law. Let’s say he will not enter the debate. (Participant 84, policymaker)

Another participant articulated how AMLO’s limited expressed commitment shows that the “face of the country doesn’t care” (Participant 1, former VAW policymaker). Thus, the perception of AMLO was that neither he nor the executive branch has taken a gender-responsive approach to policy more broadly.

More broadly, various VAW plans and policies in Mexico recognize the need to address social and gender norms around the normalization of VAW and its root causes to eradicate VAW and gender inequality (Secretaría de Salud 2020, Secretaría de Gobernación 2021). Nonetheless, participants indicated limited progress, repeatedly explaining that the greatest impediments to goals around VAW are social and cultural norms. Participants reported that gender norms, including *machismo* culture, a lack of commitment to gender equality, and the normalization of VAW, inhibited an effectively expressed commitment to ending VAW by many government actors. As some stakeholders explained, long-standing norms that do not prioritize gender equality or respect the rights of women and girls result in ineffective efforts to address VAW,

To move forward in understanding the significance of the problem we are facing, we have to recognize *machismo* … in men and women, and *machismo* culture is not going to be eliminated tomorrow. (Participant 2, civil society and former VAW policymaker)

We cannot talk about equality while we have a woman who is violated in this country because then we would not be talking about equality, and we cannot think about eliminating violence if we do not offer other alternatives such as advancing our own equality agenda, right? (Participant 8, gender policymaker)

These quotes highlight deeply entrenched social and cultural values that influence individual and institutional behavior that in turn limit political commitment more broadly and specifically limit expressed the commitment of actors within the government.

Furthermore, participants, including policymakers themselves, described government actors’ failure to see VAW as a structural issue, rather than an individual one,

Because the damages of violence are not individualized, they are social, they occur in a harmful environment that is social, part of the community, right? In other words, [the problem] is much broader, but we fail to see it in a way that is a little more comprehensive. (Participant 8, gender policymaker)

Participant 4 (VAW policymaker) also explained that violence “is reproduced due to misinformation, stereotypes, culture,” similar to others who mentioned *machismo* and the normalization of traditional gender roles and violence. A few participants also positioned VAW as a public health issue, such as Participant 85, who explained, “the problem of violence [against women] is a public health problem,” supporting her claim with the fact that the World Health Organization recognizes it as such.

In contrast, within the health sector, participants reported that greater expressed commitment to address VAW came from the MoH, particularly its National Center for Gender Equality and Reproductive Health (CNEGSR). Dr Julio Frenk (Minister of Health, 2000–06) was described by various participants as exhibiting a strong commitment to women’s health, seeking to mainstream gender into the MoH policy. While for many Dr Frenk was the most prominent champion of VAW, some participants also mentioned subsequent health ministers Dr José Ángel Córdova Villalobos (2006–12) and Dr Mercedes Juan López (2012–16) who supported VAW as a health issue, albeit in less publicly vocal ways. In 2019, the Minister of Health serving in AMLO’s cabinet, Dr Jorge Alcocer Varela, signed pronouncements focused on sexual harassment and nondiscrimination in the MoH (Centro Nacional de Equidad de Género y Salud Reproductiva 2022b), signaling that he was willing to publicly speak out against VAW against HCWs without positioning VAW as a broader societal problem.

Other health policymakers in MoH leadership positions have expressed their commitment to VAW, including Dr Karla Berdichevsky, the director of CNEGSR from 2019 to 2023. Three participants familiar with CNEGSR mentioned Dr Berdichevsky’s clear and unwavering commitment, with one participant explaining that she convened various sectors to coordinate their actions on violence prevention, taking a leadership role in these efforts (Participant 20, gender policymaker). This signaled some support to address some forms of VAW at different levels within the MoH and efforts to mainstream discussion of and policy on VAW. However, since the interviews concluded, Dr Berdichevsky left her role as director of CNEGSR, which may affect how VAW and SRH are positioned within the MoH moving forward.

### Institutional commitment

**Figure 1.**
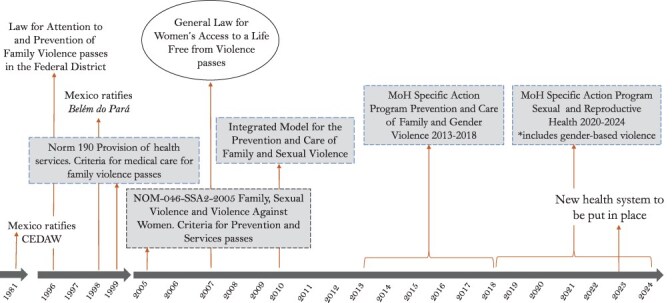
Timeline of important events in Mexico related to VAW and health.

Similar to expressed commitment, findings on institutional commitment were mixed. At the federal level, as evidenced by the document review, institutional commitment was reflected in the development of a legal and policy framework (Fig. 1) and the governance structures to implement said framework. In the document analysis, we identified a comprehensive range of laws and policies addressing VAW adopted in the 1990s and 2000s. Three landmark VAW laws include the 1996 Law for Care and Prevention of Family Violence (Mexico’s first family violence law), the 2005 Official Mexican Norm NOM-046-SSA2-2005 Family, Sexual Violence (federal health norm on VAW), and the 2007 General Law for Women’s Access to a Life Free from Violence (multi-sectoral VAW law) (Cámarade Diputados 2007; Estados Unidos Mexicanos-Secretaría de Salud 2005, Estados Unidos Mexicanos-Presidencia de la República 1998).

Although the health sector was a key sector named in the 2007 General Law, it was only in 2013 that the MoH developed its own action plan on family and gender violence, focused on reducing the associated health effects by establishing specialized medical and psychological services in health facilities (Secretaría de Salud 2014). During the presidential administration of AMLO, VAW was integrated as one of the six priorities in the 2020–24 SRH plan (Secretaría de Salud 2020), but there was no specific action plan on VAW in the health sector. One policymaker explained that this decision was made to promote cross-cutting work across SRH topics within CNEGSR (Participant 84, health policymaker). Between the 2013–18 and 2020–24 plans, many objectives and priorities remained the same, such as case detection, training, and care, but quality standards for health services for victims of VAW were added in an effort to mainstream VAW into broader sexual and reproductive health space (Secretaría de Salud 2014; Secretaría de Salud 2020).

Multiple institutions played a role in implementing policies addressing VAW (Table 4), with one specifically focusing on health: CNEGSR. The other two main institutions coordinated between various sectors, including health.

**Table 4.** Federal institutions working on gender and/or VAW in Mexico

Name of institution	Description
National Commission to Prevent and Eradicate Violence against Women (*Comisión Nacional para Prevenir y Erradicar la Violencia contra las Mujeres*, CONAVIM)	Decentralized arm of the MoI responsible for coordinating all Mexican public policies related to the prevention and response to VAW (Gobierno de México, 2021). CONAVIM also runs the National System.
National Institute of Women (*Instituto Nacional de las Mujeres*, INMUJERES)	Decentralized public entity under the Federal Public Administration that addresses gender issues more broadly than CONAVIM, but also works on VAW.
National Center for Gender Equality and Reproductive Health (*Centro Nacional de Equidad de Género y Salud Sexual y Reproductiva*, CNEGSR)	Located within the MoH, since 2003 the CNEGSR has been responsible for health policy and service implementation regarding IPV.

While establishing institutions is imperative, what matters for implementation is the work of civil servants inside of institutions. Participant 19, a health policymaker, highlighted some institutional challenges that civil servants experienced,

We observe the apathy of the people who serve at the public level, right? Unfortunately, you also have to know that even [people] dedicated to addressing the issue are not properly trained and can reproduce many belief systems that label women as responsible and guilty of continuing to receive violence. That is extremely worrisome. (Participant 19, health policymaker)

Some participants also highlighted that the lack of political will at the higher policy level (presidency) influences the implementation of VAW policies, as some institutions had their work and strategies minimized. One policymaker working on gender explained, “unfortunately, you do not find the same will to implement [VAW] in all institutions” (Participant 18, gender policymaker). Another explained, “Institutions that work on this issue have been minimized, their strategies have changed, that is at the level of the head of the country, as there is no political will” (Participant 1, former VAW policymaker). A policymaker who had worked in the government shared that within the Ministry of the Interior (MoI), “[VAW] is not a topic of interest” (Participant 22, former VAW policymaker). Relatedly, one participant mentioned that CONAVIM (the National Commission to Prevent and Eradicate Violence Against Women), located in the MoI, only had 40 employees, an insufficient number to adequately run the Commission (Participant 4, VAW policymaker).

Under the General Law, each presidential administration must publish its own comprehensive multi-sectoral VAW program describing each sector’s programmatic priorities. However, according to various participants, under AMLO’s administration the VAW program (Secretaría de Gobernación 2021) was published 3 years late due to “a budget issue, it got stuck and the Treasury wasn’t approving it” (Participant 22, former VAW policymaker). As a result, there was no clear guidance/regulations on programs and priorities emanating from the federal government for VAW for 3 years, and the health sector could not develop a VAW plan between 2018 and 2020.

According to some participants, Mexico’s system for monitoring the implementation of the General Law and the aforementioned administration-specific plans was not working as designed. The National System to Prevent, Respond to, Sanction, and Eradicate Violence Against Women (hereafter “National System”) was reported to lack prominence, with meetings that were not fruitful because they were too high level and focused more on political pronouncements rather than concrete actions. Participants expressed a disconnect between those who were part of the system and attended meetings and those responsible for implementing and operating programs. For example, one meeting attendee shared, “I do not think it is necessarily that [the National System] does not work at all … I believe that the institutions represented are not necessarily the institutions that operate the most important programs around violence” (Participant 82, government researcher). This was echoed by a gender policymaker (Participant 20) who identified a lack of governance structure or infrastructure for health services for VAW.

When describing the limited implementation of policies to support survivors, health managers highlighted the normalization of violence, lack of recognition of VAW as a health issue, and patriarchal gender norms within the medical community. One participant articulated how gender stereotypes were reproduced in the consultation rooms of psychologists (Participant 6, civil society actor). Participants noted that traditional and patriarchal attitudes in medical discourse have historically devalued women and emphasized the need to challenge and change these normalized perceptions to effectively address violence,

I believe that society and doctors, in general, normalize violence because we are living in a violent environment in the country, so listening to thirty deaths here, fifteen there, well, one ends up normalizing it. (Participant 40, health facility manager)

… many things are normalized, such as violence against women and violence of all kinds, that is, verbal, psychological, many things we think are normal, so I think we have to take away that idea of normalizing violence, and by taking away that idea, obviously you are going to solve it, like the whole issue. (Participant 51, health facility manager)

Policymakers committed to gender equality emphasized the need to address violence in the health system, especially at health facilities, and the institutions tasked with policy implementation. An NGO leader described a course for public officials, including HCWs, focusing on personal behavior transformation to prevent and identify violence (Participant 6, civil society actor). Another policymaker stressed the importance of training and sensitizing healthcare providers to eliminate the normalization of violence and harmonize laws and regulations related to women’s health (Participant 14). These insights reflect a broader societal desensitization to violence, which participants connected to the weak implementation of institutional strategies within the health system to end VAW.

### Budgetary commitment

At the federal level, Mexico had a clear budgetary commitment to address VAW as part of its broader strategy on gender equality. In 2003, the Commission on Equity and Gender in the House of Representatives began to advocate for the reallocation of resources for women’s issues, including VAW; however, it was not until 2007, with a new government and legislation in place that two line items related to gender were added to the federal budget (Participant 85, former VAW policymaker). The federal gender budget, which we analyzed as part of our document review, increased steadily between 2008 and 2015 (growing 17.2% over that period), including specific indicators on eradicating VAW (Centro de Estudios para el Adelanto de las Mujeres y la Equidad de Género 2015). In 2019, 260.5 million pesos (∼12.2 million USD) were designated to promote prevention and care for VAW (Centro de Estudios de Finanzas Públicas 2019).

While the federal government has allocated a VAW budget, participants questioned whether it has been sufficient and how it has been used. The VAW budget is wrapped into the broader gender budget, suggesting a greater focus on gender issues than on the health system’s response to VAW. Before the CNEGSR was created, there was a budget for gender (including VAW), but it was difficult for the resources allocated by the Minister of Health to be used for VAW,

… there were always other characters … who decided “good, the legislature assigned 5 million pesos for Women and Health. Well, the Women’s Hospital needs some of those 5 million pesos for who knows what”… But that’s not what the money was for. It was for gender mainstreaming, but we had to fight like that, almost slapping people away saying, “leave that money.” (Participant 85, former VAW policymaker)

Several participants mentioned the availability of a specific MoH budget, indicating progress in addressing VAW as a health issue. However, a few doubted whether VAW would continue to be funded in health. Notably, although the MoH has financed specialized services for VAW since the 2000s, there is no guarantee that the program will continue to receive financial resources. A policymaker working on VAW at the MoH revealed there have been internal discussions about whether it should continue,

… within the Ministry [of Health], we have discussed whether specialized services are necessary or not and, if the budget is reallocated, how we run the risk of not being able to serve patients. For Karla [CNEGSR director], the risk has been very clear, and she has been able to position it within the broader context. However, when decisions are made hierarchically, we need to ensure that violence is included, like tuberculosis and vaccination. (Participant 84, SRH policymaker)

Following budget allocations to expenditures was beyond the scope of our study, but some participants expressed they felt the budget was insufficient, and a few participants indicated a lack of funding as the greatest challenge Mexico faces regarding VAW. A related concern was whether the budget has produced results. An official federal audit of CONAVIM (Auditoría Superior de la Federación 2019) highlighted the disconnect between the budget and the institutional structures to implement the policy:

The budget design was not consistent with the public problem because the policy does not have a comprehensive programmatic structure that includes the total number of branches, functions and sub-functions, budget programs, and responsible units through which the resources can be used to operate and steward programs and policies. (Auditoría Superior de la Federación 2019, p. 87)

The audit also revealed that in 12 years between the time that the General Law was passed and when the audit took place, there was only one plan in place for 5 years defining the responsibilities of different federal authorities, and in some cases, the authorities did not have adequate resources to meet those commitments (Auditoría Superior de la Federación 2019). Another report showed that in 2021, the federal spending on programs to prevent and respond to VAW was less than assigned, with 15% of the budget remaining unused (Ruiz 2022).

## Discussion

These findings demonstrate that while Mexico has made significant progress toward addressing VAW on paper, many barriers to sustained political commitment and implementation remain. Past research on shortfalls in implementing VAW legislation can broadly be grouped into political or legal, logistical, financial, human resources, and cultural categories, and our findings reflect challenges in many of the same categories (Hughes 2017). Although few studies have been conducted on the implementation of VAW health policies in Latin America specifically, our findings are similar to those from Brazil where, despite a comprehensive policy and legal framework on VAW, this issue has not been given political support, causing problems in the health system’s response to domestic violence (d’Oliveira et al. 2020).

In this discussion, we contextualize our findings within the framework of the Mexican presidency and the broader executive, the role of individual champions as well as institutions, and the implications of the utilization of VAW/gender budgets. We then explore the mediating role of values, contextualizing our findings within recent shifts to consider values more explicitly in health policy and systems research. Finally, we outline policy implications and potential solutions and discuss various limitations of the study.

### Mixed efforts from the executive to address VAW

AMLO’s “Fourth Transformation” was intended to chart a more progressive direction, but he showed little concern with gender equality and spoke against feminists and survivors of IPV (Pedroza 2019, Sánchez Talanquer 2020). We found that AMLO’s executive branch (during the 2018–24 presidential administration) had not demonstrated expressed commitment, which is significant because Mexico has a strong presidential system with wide-ranging powers (Mainwaring and Shugart 1997), and power is concentrated in the executive branch. The position of the president on issues such as VAW is crucial because many policies are designed to be implemented using a top-down approach (Parkhurst and Lush 2004, Youde 2007, Baker et al. 2018). The president’s absence of expressed commitment may negatively impact tangible areas of policymaking, such as institutional and budgetary priorities. Furthermore, participants described how cultural norms and values mitigate other government stakeholders’ expressed commitment to ending VAW. This underscores that the dimensions of political commitment we discuss are connected and reinforce and/or weaken each other in important ways.

In contrast, the expressed commitment from the MoH to address VAW over time was significant, including the role that the director of CNEGSR played to position VAW as an important SRH issue within the MOH. Her role is consistent with research on how “organizational champions” can help implement health system changes (Hendy and Barlow 2012, Santos et al. 2022). These champions are critical in low- and middle-income contexts where human resource issues can constrain policy implementation.

### Weakening institutions in Mexico

Mexico’s VAW policy framework signals that some priority has been given to this issue (World Health Organization 2021a). Yet, the institutions responsible for implementing these laws and policies were not up to the task, as other studies have also noted (Htun and Jensenius 2020). Limited support from the president affected how institutions operated and whether they could ensure effective policy implementation. Beyond a lack of expressed or symbolic leadership in this area, AMLO has also been strengthening presidential power and dismantling and weakening autonomous institutions, including those that work on gender and VAW (Dresser 2022). At the same time, institutional priorities are shaped by societal norms and values and institutional commitment to ending VAW is weak, as seen by the limited recognition by medical professionals that VAW is a health issue rather than a private one. Taken together, it seems that institutions were not exhibiting the commitment necessary to fully implement laws and policies for VAW.

### A complicated picture of how the budget is used

Budget allocations have been recognized as crucial to the health system’s response to VAW because they allow for the training and support of front-line HCWs (García-Moreno et al. 2015b). While funding for VAW has been allocated through several Mexican administrations, it was often wrapped into a wider gender budget, leading to concerns about how the budget was used and whether it produced the intended results. Deficiencies and cuts to financing for VAW in Mexico noted elsewhere (CEPAL 2013, Solis et al. 2022) have been labeled a type of “institutional *machismo*” (Ruiz 2022) and likely also affect how institutions operate.

### Cultural and normative factors mediate political commitment in a country with widespread machismo

Our results underscore that values, particularly cultural and normative factors, are a crucial mediator for the political commitment to address VAW in health policy and political decision-making (Whyle and Olivier 2020, Buse et al. 2023). Values have been shown to affect policy implementation in other settings, such as adopting interventions to address IPV in the occupied Palestinian Territory and the health system’s readiness to respond to VAW in Brazil (Colombini et al. 2020, 2022, 2024). Research on agenda-setting in Nepal shows that only certain ways of framing the problem (e.g. as a human rights issue) successfully got VAW on the policy agenda, highlighting the importance of how this issue is viewed and defined (Colombini et al. 2015). However, despite the different ways of defining the problem, gender-unequal values and social norms were perceived to be the greatest challenge to addressing VAW across participants, reflecting the gendered dimension of this problem, which is consistent with other literature on gender inequality in health and health system (Heise et al. 2019, Crespí-Lloréns et al. 2021).

Existing research has shown that after the passage of the General Law, the share of women experiencing IPV declined, reporting rates rose, attitudes condoning violence shifted, and women learned about legislation to protect their rights, indicating how legislation has the potential to shift social norms (Htun and Jensenius 2022). While that study and other studies have shown that gender norms are changing in Mexico (Agoff and Espinoza Cid 2020, Htun and Jensenius 2022), our findings show that society writ large has yet to fully embrace the rights and principles included in Mexico’s comprehensive legal framework for VAW around the need to address and eliminate violence, and that values and norms may affect whether existing institutions can implement policies successfully. This echoes research by Htun and Jensenius (2020), who posit that Mexican laws addressing VAW are aspirational, and VAW is still normalized in Mexican society and in the health system, as shown elsewhere (Herrera 2010, Martínez Rivera et al. 2021).

Gore et al. (2014) flagged that states’ commitment to service provision—in the context of HIV care—may be influenced by subjective aspects, namely those that are normative (i.e. protection of human rights) and ideological (i.e. decisions driven by evidence). We show that norms and ideology, especially regarding gender norms and cultural attitudes, can play an influential role in mediating the three dimensions of political commitment, and in this case, values came through as strongly mediating institutional and expressed commitment.

As this case shows, while the laws supporting VAW are quite strong in Mexico, implementation falls short. Both expressed and institutional commitment were limited by the normalization of VAW. A direct policy implication is that it is critical to increase sensitization among HCWs caring for survivors seeking health care as a result of experiencing violence. At the same time, broader societal efforts to end VAW, destigmatize survivors who seek care, and promote gender equality could improve outcomes for survivors and also help shift societal norms, which could improve political commitment. Evidence shows that strongly designed and implemented interventions focused on multi-year community activism efforts can shift harmful gender attitudes, roles, and social norms but will require commitment, investment, and time (Kerr-Wilson et al. 2020).

## Limitations

This study has some limitations. First, while we focused on analyzing the implementation of Mexico’s VAW policy using the political commitment framework, alternative frameworks could offer valuable insights. In our findings, values came through so strongly that there is a possibility that they are acting as more than a mediator, and future research should build on Fox et al. (2011) framework to look at values as their own dimension of political commitment. Furthermore, while our key informants regularly identified values, norms, and culture as the most important mediators of the three dimensions of political commitment, we cannot and should not rule out that other factors are also important mediators. Second, although the study identified conditions for sustained political commitment, it did not examine concrete policy outcomes, which should be a focus of future research. Finally, interviews were conducted solely in Mexico City, excluding the perspective of state-level policymakers and actors. Moreover, some policymakers reported limited experience with the health system. However, this led to broader insights than anticipated, which—given their roles at national-level organizations—allowed for understanding dynamics beyond the capital but may limit generalizability beyond Mexico.

## Conclusion

Addressing VAW presents unique challenges because there are many short- and long-term consequences for women’s lives, implicating various actions and sectors in the necessary response (Aguerrebere et al. 2021, World Health Organization 2021b). Mexico passing a comprehensive legislative and policy framework to respond to VAW and then lagging on implementation provides a helpful example of the challenges that countries in Latin America and other low- and middle-income contexts are facing regarding VAW, a persistent and complex problem. This case underscores how the passage of laws/policies, the existence of institutions, and the budget are necessary but not sufficient conditions for successful implementation, and values that mediate political commitment are critical and often overlooked contributing factors to success. Our approach to studying implementation through the lens of political commitment enhances our understanding of how health issues requiring a multi-sectoral response are implemented over years and decades and may be useful for others studying a range of policy issues across contexts. Sustained political commitment is crucial for implementation, especially in a context like Mexico, where laws and policies have existed for decades.

## Supplementary Material

czaf012_Supp

## Data Availability

All policy documents analyzed are publicly available online. The interview data cannot be shared publicly due to privacy concerns regarding the participants and the sensitive nature of the topic. The corresponding author will share the de-identified interview data upon reasonable request.

## Reflexivity statement

The authors believe that to decolonize global health research in low- and middle-income settings, it is vital to recognize local research assistants’ often hidden roles and to collaborate with regional experts. Two coauthors are Mexican medical students who worked as research assistants on this project and were involved in data analysis throughout the project. More recently, they have presented the findings of this work at an academic conference on gender-based violence in Mexico City. One coauthor is a senior researcher on health at a top public institution in Mexico City who was involved with the study design and supported data collection efforts. The non-Mexican authors have extensive research experience in Mexico and its health system and have worked in various LMIC settings on the health system response to VAW. Moreover, the initial findings were shared with Mexican civil society organizations and academics to get feedback throughout the analysis process. All of the authors of this paper identify as cisgender women and are based in the USA, Mexico, and the UK. We all have a clear position opposing VAW in all its forms and strive to advance the health system response to improve the health outcomes of survivors of violence across Latin America and the globe.
