# Supplementary material for: Political commitment and implementation: the health system response to violence against women in Mexico
Source: Health Policy Plan. 2025 Mar 8;40(5):519–30. doi: 10.1093/heapol/czaf012 (PMC12063586; doi:10.1093/heapol/czaf012)
Supplement: czaf012_Supp [file czaf012_supp.zip › Table 5.docx]

**Table 5. Policy documents analyzed**

| **National legal framework** | |
| --- | --- |
| **Document Name** | **Year(s)** |
| *Ley General de Acceso de las Mujeres a una Vida Libre de Violencia* | 2007 |
| *Reglamento de la Ley General de Acceso de las Mujeres a una Vida Libre de Violencia* | 2015 |
| **National policies and plans** | |
| **Health Policies and Plans** | |
| **Document Name** | **Year(s)** |
| *Programa de Acción Específico* | 2020-2024 |
| *Manual de Organización Específico del Centro Nacional de Equidad de Género y Salud Reproductiva* | 2012 |
| *Programa de Acción Específico Prevención y Atención de la Violencia Familiar y de Género 2013-2018* | 2013-2018 |
| *Norma Oficial Mexicana, NOM-046-SSA2-2005.Violencia familiar, sexual y contra las mujeres. Criterios para la prevención y atención.* | 2005 |
| **Violence Against Women and Gender Policies and Plans** | |
| **Document Name** | **Year(s)** |
| *PROGRAMA Integral para Prevenir, Atender, Sancionar y Erradicar la Violencia contra las Mujeres 2021-2024* | 2021 |
| *Programa Integral para Prevenir, Atender, Sancionar y Erradicar la Violencia Contra las Mujeres 2014-2018* (Comprehensive | 2015 |
| *Programa Integral para Prevenir, Atender, Sancionar y Erradicar la Violencia Contra las Mujeres* | 2010-2012 |
| *Programa Institucional 2020-2024 del Instituto Nacional de las Mujeres* | 2019-2024 |
| *Ley de Acceso de las Mujeres a Una Vida Libre de Violencia del Distrito Federal* | 2008 |
| **Budget documents** | |
| **Document Name** | **Year(s)** |
| *Presupuesto Público con Perspectiva de Género versus Recursos Federales Etiquetados en México para la Igualdad entre Mujeres y Hombres, 2008-2019* | 2019 |
| *Presupuestos Públicos con enfoque de género en México* | 2018 |
| *Políticas Públicas y Presupuesto Etiquetado para la Igualdad entre Mujeres y Hombres en México* | 2015 |
